# The Effect of a Low Glycemic Index Pulse-Based Diet on Insulin Sensitivity, Insulin Resistance, Bone Resorption and Cardiovascular Risk Factors during Bed Rest

**DOI:** 10.3390/nu11092012

**Published:** 2019-08-27

**Authors:** Ruirui Gao, Whitney Duff, Donna Chizen, Gordon A. Zello, Philip D. Chilibeck

**Affiliations:** 1 College of Kinesiology, University of Saskatchewan, Saskatoon, SK S7N 5B2, Canada; 2 College of Medicine, University of Saskatchewan, Saskatoon, SK S7N 5B2, Canada; 3 College of Pharmacy and Nutrition, University of Saskatchewan, Saskatoon, SK S7N 5B2, Canada

**Keywords:** bed rest, pulse, insulin sensitivity, insulin resistance, bone resorption, cardiovascular risk factors

## Abstract

We determined the effects of a low glycemic-index pulse-based diet (i.e., containing lentils, chick peas, beans, and split peas) compared to a typical hospital diet on insulin sensitivity assessed by the Matsuda index from the insulin and glucose response to a two-hour oral glucose tolerance test, insulin resistance assessed by the homeostatic model assessment of insulin resistance (HOMA-IR), bone resorption assessed by 24 h excretion of urinary n-telopeptides(Ntx) and cardiovascular risk factors (blood lipids, blood pressure, arterial stiffness and heart rate variability) during bed rest. Using a randomized, counter-balanced cross-over design with one-month washout, six healthy individuals (30 ± 12 years) consumed the diets during four days of bed rest. The Matsuda index, HOMA-IR, urinary Ntx and cardiovascular risk factors were determined at baseline and after the last day of bed rest. Compared to the typical hospital diet, the pulse-based diet improved the Matsuda index (indicating increased insulin sensitivity; baseline to post-bed rest: 6.54 ± 1.94 to 6.39 ± 2.71 hospital diet vs. 7.14 ± 2.36 to 8.75 ± 3.13 pulse-based diet; *p* = 0.017), decreased HOMA-IR (1.38 ± 0.54 to 1.37 ± 0.50 hospital diet vs. 1.48 ± 0.54 to 0.88 ± 0.37 pulse-based diet; *p* = 0.022), and attenuated the increase in Ntx (+89 ± 75% hospital diet vs. +33 ± 20% pulse-based diet; *p* = 0.035). No differences for changes in cardiovascular risk factors were found between the two diet conditions, with the exception of decreased diastolic blood pressure during day three of bed rest in the pulse-based versus hospital diet (61 ± 9 vs. 66 ± 7 mmHg; *p* = 0.03). A pulse-based diet was superior to a hospital diet for maintaining insulin sensitivity, preventing insulin resistance, attenuating bone resorption and decreasing diastolic blood pressure during four days of bed rest.

## 1. Introduction

Bed rest is sometimes a necessary component of patient care during recovery from medical conditions. Bed rest is associated with unweighting the body and after only a short period of time (3–4 days), raises blood glucose and insulin, promotes bone loss, and increases arterial stiffening [1,2,3]—conditions that are also synonymous with low physical activity levels. The maladaptive glucose changes associated with bed rest have important clinical implications during hospital stays that include increased mortality, morbidity, length of hospital stay, and health care costs [4,5,6,7,8].

Detrimental outcomes with bed rest may be exacerbated or mitigated through diet. With an aim to prevent muscle and strength loss, studies of dietary interventions typically employ animal-derived high-quality protein diets [9,10]. However, animal-based diets may exacerbate: (1) impaired glucose regulation by reducing blood glucose clearance via acylcarnitine (metabolic by-products of branch-chain amino acid catabolism)-induced inhibition of insulin receptors at the muscle fibre membrane [11]; (2) bone mass loss [12] because bone breaks down to release bicarbonate to buffer high blood acidity induced by cationic and sulphur-based amino acids that are contained in animal proteins; and (3) arterial stiffness, a major risk factor for cardiovascular disease (CVD) because high blood glucose and insulin levels promote arterial wall damage [13]. The typical hospital diet contains many animal proteins and high glycemic index foods that induce higher and faster increase in blood glucose concentration and insulin release. Pulses (i.e., lentils, chickpeas, peas, beans) have a low glycemic index and have been used in clinical populations to improve insulin sensitivity and reduce insulin resistance [14,15]. They also contain high quality proteins that have lower levels of sulphur-based amino acids and therefore may be superior for preventing bone resorption. Dietary interventions during bed rest studies have mainly been aimed at prevention of muscle and strength loss; no studies focusing on prevention of blood glucose increase or cardiovascular problems have been conducted.

The purpose of this study was to determine the effects of a pulse-based diet compared to a hospital diet on insulin sensitivity, insulin resistance, bone resorption, CVD risk factors (i.e., blood lipids, blood pressure, arterial stiffness and heart rate variability) and body composition during four days of bed rest. We hypothesized that a pulse-based diet would be superior to a hospital diet for preventing loss of insulin sensitivity, insulin resistance, and bone catabolism, and reducing CVD risk during four days of bed rest.

## 2. Methods

### 2.1. Participants

Six healthy adults (2 males, 4 females, 30 ± 12 years, BMI 23.3 ± 4.5 kg/m^2^) participated in this study. Inclusion criteria were as follows: 18 years of age or older, not diabetic, not on medications that affect carbohydrate metabolism or cholesterol levels, not vegetarian, and not allergic to lentils, beans, chickpeas, peas, or meats, and do not have high blood pressure (>144/94) or other hematological complications (deep vein thrombosis, clotting issues). All participants signed an informed consent form. This study was approved by the University of Saskatchewan Biomedical Research Ethics Board (reference number Bio#17-176). ClinicalTrials.gov Identifier: NCT03595943

### 2.2. Study Design

**Figure 1:**
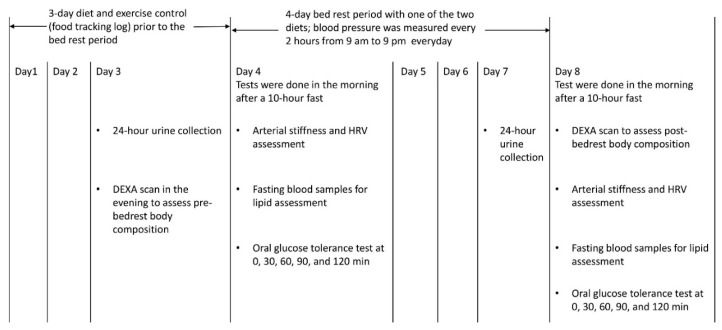
Study design. Abbreviations: DEXA, dual energy X-ray absorptiometry; HRV, heart rate variability.

The study used a counterbalanced, double-blind, cross-over design where participants were randomized to two conditions: (1) four days of bed rest while on a pulse-based diet (i.e., meals and snacks containing lentils, beans, chickpeas, and peas); or (2) four days of bed rest with a hospital diet (i.e., containing protein sources from animal products) based on the regular patient menu from our university hospital (Royal University Hospital). Participants underwent bed rest for four days with one of the two diets in our research laboratory, had one-month wash-out period and then returned to the lab for another four-day period of bed rest with the other diet (Figure 1). During the bed rest period, participants were allowed to read, do computer work, or watch television. Participants were allowed washroom breaks as needed and were supervised by two investigators 24 h per day while undergoing the four days of bedrest.

### 2.3. Measurements

Resting metabolic rate. At least one week before the bed rest period, tests for resting metabolic rate were performed as previously described [16]. This was used to determine the amount of food during the bed rest phases. The total amount of calories for the meals per day was 1.1 times resting metabolic rate to account for the thermic effect of food or the calories required to digest and absorb food [17].

Body composition. The evening before and the morning after each bed rest period, dual energy X-ray absorptiometry (QDR Discovery Wi, Hologic Inc., Bedford, Maryland, USA) and QDR software for Windows XP (QDRDiscovery, Hologic, Inc) was used to measure body composition: bone mineral content (BMC), fat mass, lean mass, lean mass + BMC, total mass, %fat, bone mineral density (BMD), trunk fat mass, spine BMC, spine BMD, total hip BMC, total hip BMD, femoral neck BMC and femoral neck BMD were assessed. The coefficients of variation for these measurements from our lab range from 0.5% (whole body BMD) to 3.0% (whole body fat mass) [18].

Bone resorption. The day before the bed rest periods and the last day of each bed rest period, 24-h urine was collected in a plastic container for the measurement of N-telopeptides of bone type I collagen (Ntx) and creatinine (for Ntx correction). Urine collection and urine sample analysis were conducted as previously described [19]. Briefly, urine samples were analyzed with creatinine colorimetric assay kits (Cayman Chemical Company, Ann Arbor, MI, USA) and Osteomark^®^ NTx Urine assay kits (Alere Scarborough, Inc., Scarborough, ME, USA). The intra-assay coefficient of variation for the assessment was 9.4% [19]. The amount of NTx per day for each individual was calculated by multiplying the concentration by the 24-h urine volume [19]. Lean tissue mass was also used for NTx correction [19].

Blood samples. After a 10-h overnight fast, the morning of the first day of each bed rest period and the morning following the final night of bed rest, a 75-g oral glucose tolerance test was used to determine the total area under the blood response curve (TAUC, including the baseline) and the incremental area under the curve (IAUC, excluding the area beneath the baseline level) for glucose and insulin. Blood samples were collected at baseline, 30, 60, 90 and 120 min after beverage consumption from an antecubital vein. Homeostatic model assessment of insulin resistance (HOMA-IR) was calculated using the formula: HOMA-IR = [fasting plasma glucose (mmol/L) × fasting plasma insulin (µIU/mL)/22.5] [20]. The Matsuda index was calculated to assess whole-body insulin sensitivity using the formula: Matsuda index =10,000 /√Gf × 18 × If ×Gm × 18 × Im, where Gf is fasting plasma glucose concentration (mmol/L), If is fasting plasma insulin concentration (µIU/mL), Gm is mean plasma glucose concentration during oral glucose tolerance test (mmol/L), and Im is mean insulin concentration during oral glucose tolerance test (µIU/mL) [21]. Fasting blood samples were also collected to assess lipids: total cholesterol, triglycerides, high density lipoproteins (HDL), low density lipoproteins and very low density lipoproteins (LDL + vLDL). Blood samples were analyzed as described in our previous study [16]. The intra-assay coefficient of variation for the insulin assay were <3.5% and <4% for all the noninsulin assays [16].

Arterial stiffness, heart rate variability (HRV) and blood pressure. On the same days and at the same time for pre- and post-testing prior to the oral glucose tolerance test, HRV was determined following 10 min of supine rest from a 5-min surface electrocardiogram recorded epoch (Bio Amp, ADInstruments Bella Vista, New South Wales, Australia). Arterial stiffness was measured by pulse wave velocity with applanation tonometry (Mikro-tip Catheter Transducer model SPT-301, Millar Instruments, Inc, Houston, TX, USA) at the carotid and femoral sites using the foot-to-foot technique [22]. Pulse wave velocity was computed as D/Δt, where D was the distance between carotid and femoral sites, and Δt was the time between these two pulse waves. Signals were converted from an analogue to a digital signal (PowerLab 16/35, ADInstruments Bella Vista, New South Wales, Australia) and analyzed using LabChart (ADInstruments Bella Vista, New South Wales, Australia). Blood pressure was measured every 2 h from 09:00 to 21:00 during each 4-day period of bed rest (Welch Allyn Spot Vital Signs Monitor, 4200B, Welch Allyn Ltd., NY, USA). A single blood pressure recording every 2 h was an average of three recordings. A blood pressure recording for each day was an average of all the recordings over the course of 12 h.

### 2.4. Diets during Bedrest Period

Diets were prepared in our food laboratory in the College of Pharmacy and Nutrition, University of Saskatchewan. The pulse-based diet was prepared based on a variety of recipes from our previous intervention studies [15,23] that successfully improved blood pressure, insulin levels, and blood lipid profile in clinical populations. The hospital diet was based on the patient menu from the Royal University Hospital (Food and Nutrition Services, Saskatoon Health Region) and contained foods with a mixture of glycemic indexes, but many with higher glycemic index such as rice krispies, crackers, muffins, and meals derived from animal proteins (i.e., roast turkey and beef). The amount of calories delivered from the pulse-based diet was matched with that from the hospital diet, as was protein, carbohydrate, and fat content (Table 1).

### 2.5. Diet and Exercise Control Prior to Bed Rest Period

Participants were asked to keep their routine physical activities and regular diet that contained at least 150 g of carbohydrate per day for three consecutive days prior to the bed rest period. They were also asked to refrain from smoking and consuming any alcohol or caffeinated drinks during these three days. They kept a food diary during these three days and repeated the same diets 3 days prior to the second condition.

### 2.6. Statistical Analyses

Statistical analyses were performed using SPSS (SPSS Version 21; SPSS Inc., Chicago, DE, USA). All data except for blood pressure were assessed by determining change scores from baseline to after the four-day bed rest periods and then comparing the change scores between diets with a one-factor repeated-measures analysis of variance. Blood pressure was assessed by two-factor (diet and time) repeated measures analysis of variance. A Bonferroni post-hoc test was used to test for differences between means when there was an interaction for the blood pressure analysis. Data are presented as means ± SDs. Alpha level was set at 0.05.

## 3. Results

### All Six Participants Tolerated the Bedrest Condition and No Participants Dropped Out

Change scores for each outcome for both conditions are shown in Table 2. Compared with the hospital diet, the pulse-based diet elicited a greater increase in the Matsuda Index (*p* = 0.017), a greater decrease in HOMA-IR (*p* = 0.022), and a smaller increase in absolute Ntx level (*p* = 0.035) and Ntx/lean tissue mass (*p* = 0.042). No condition differences were found in change scores for other outcomes in Table 2.

Time main effects for all the outcomes are shown in Table 2. Post-bedrest glucose I-AUC, glucose T-AUC, HOMA-IR, absolute Ntx level, Ntx/lean mass, lean mass, lean + BMC, total mass, % fat, total hip BMD and LF/HF were significantly different from pre-bedrest (*p* < 0.05).

There was a significant diet × time interaction for diastolic blood pressure (*p* = 0.042, Table 3). Diastolic blood pressure was lower on day 3 while on the pulse diet compared to the hospital diet. No differences were found between other blood pressure outcomes.

## 4. Discussion

The major finding of this study was that a four-day pulse-based diet during bed rest induced a significant increase in the Matsuda Index compared to a typical hospital-based diet, indicating that insulin sensitivity was improved in the pulse-diet phase compared to the hospital-diet phase. In addition, HOMA-IR was reduced to a greater extend with the pulse-based diet, indicating that the pulse-based diet was better than the hospital diet for reducing insulin resistance. Ntx increased to a smaller extent on the pulse-based diet than the hospital diet, indicating that the pulse-based diet attenuated bone resorption. Diastolic blood pressure was lower on day 3 during the pulse diet compared to the hospital diet. This is the first study to assess a dietary intervention to prevent the reduction in insulin sensitivity and increase in insulin resistance during bed rest.

### 4.1. Effect of Pulse-Based Diet on Insulin Sensitivity and Insulin Resistance

In the present study, a significant condition difference was found for the Matsuda Index and HOMA-IR, indicating that a pulse-based diet, compared with typical hospital diet, improved insulin sensitivity and prevented insulin resistance during 4-day bed rest. No difference was found between conditions for the glucose and insulin AUC.

The improvement in insulin sensitivity/resistance in the pulse-based diet condition might be due to the low glycemic index, high fiber content, high content of micronutrients, and low sodium content in pulse diet. Pulses have a low glycemic index, which tends to induce lower rate of glucose increase and insulin secretion, whereas the higher glycemic index hospital diet might promote insulin resistance through stimulation of glucose release and insulin secretion. High glucose induced by high-glycemic index foods could down-regulate some aspects of insulin signaling, e.g., the CAP-Cbl signaling pathway [24]. Insulin inhibits fat oxidation and stimulates fatty acid synthesis [25]. This fat accumulation could be associated with increased insulin resistance with down-regulation of post receptor signaling and insulin receptors [26]. Also, pulses are rich in fiber and an increased fiber intake is associated with lower insulin resistance [27] via interference with the absorption of dietary protein [28]. Infusion of animal-based amino acids induces insulin resistance with phosphorylation of downstream factors involved in insulin-signaling cascade [29] and therefore animal-protein diets, e.g., the hospital diet, may increase insulin resistance and diabetes risks [29]. Furthermore, pulses are rich in micronutrients, such as zinc and selenium, which might be of potential importance. Zinc, concentrated in the pancreas, is important in insulin secretion and biosynthesis via the activation of phosphatidylinositol protein kinase B and protein 3-kinase, and stimulation of insulin receptor (β-subunit) phosphorylation [30,31]. Higher zinc intake reduces insulin resistance [31] while zinc deficiency results in insulin resistance and glucose intolerance [32]. Selenium might affect insulin resistance via multiple routes including oxidative stress, inflammatory cytokines and insulin-like action [33]. Higher dietary selenium intake has been reported to induce lower insulin resistance [33,34,35]. The low sodium content in the pulse diet might be a final factor that lowers insulin resistance. Some studies suggest that a low sodium diet might decrease insulin resistance by reducing blood leptin levels [36], bradykinin concentration [37] and angiotensin II levels [38].

### 4.2. Effect of Pulse-Based Diet on Bone Resorption and Body Composition

We found that the pulse-based diet attenuated the increase in Ntx during 4-day bed rest, compared with the typical hospital diet. No difference was found for changes in body composition between conditions. The beneficial effects of pulse-based diet on bone resorption might be explained by the differences in amino acid profile between two types of diets. Typical hospital diets contain protein from animal sources which stimulate bone catabolism [12]. Animal-based proteins contain cationic and sulphur-based amino acids that result in the acidification of blood. Bone serves as a reservoir of bicarbonate, which helps to buffer acidity in blood. When blood acidity is high, bone breaks down to release bicarbonate, eventually weakening bone. Indeed, the latest generation of bed rest studies that use whey protein to prevent muscle loss also include supplementation with an alkalinizing agent to prevent this metabolic acidosis [39]. Pulses have high quality proteins that do not induce metabolic acidosis and have lower levels of sulphur-based amino acids (i.e., methionine and cysteine) compared to meat-based proteins such as turkey and beef [40]. As such, they may be of benefit for preventing breakdown of bone during bed rest. The different sodium content might also affect bone resorption. High sodium chloride intake increased bone resorption by increasing urinary calcium excretion [41] and parathyroid hormone secretion [42] which stimulates the release of calcium from bone.

### 4.3. Effect of Pulse-Based Diet on CVD Risk Factors

In the current study, diastolic blood pressure was lower on day three while on the pulse diet compared to the hospital diet. No differences were found between conditions for changes in other cardiovascular risk factors (i.e., blood lipids, arterial stiffness and HRV). We hypothesized that the pulse-based diet, compared with typical hospital diet, might prevent deterioration of CVD risk factors during 4-day bed rest because of the high content of fiber, low-glycemic index, effects on gene regulation, low sodium content and a high isoflavone content. Dietary fiber increases the excretion of bile acid and reduces reabsorption of bile acids, thus lowering cholesterol levels [43,44]. Soluble dietary fiber also produces short-chain fatty acids, which potentially affects fatty acid metabolism and cholesterol synthesis [45,46]. Low-glycemic index food induces slower and lower increase of glucose and insulin, a hormone that inhibits fat mobilization and oxidation [25], thus potentially increasing fat oxidation and reducing harmful blood lipid levels. Fat build-up in blood vessels is an important and key precursor to arterial stiffening and cardiovascular disease [47]. In contrast, insulin and glucose levels increase faster and greater after high-glycemic index food consumption. Hyperinsulinemia and hyperglycemia induce oxidative stress (which damages arterial walls) [48], reduces nitric oxide (which is a powerful vasodilator and helps reduce inflammation) [24] and finally leads to arterial wall damage and arterial stiffness [13], meaning that low-glycemic index pulse-based foods have the potential to prevent arterial stiffening, a major risk factor for cardiovascular disease [49]. Pulses might further reduce harmful blood lipid levels by up-regulating genes involved in acetyl-CoA degradation and β-oxidation, and down-regulating genes related to lipogenesis and glycolysis [50]. In the current study, the reduced diastolic blood pressure on the pulse diet might be due to the lower sodium content of this diet. This is supported by many studies which reported that a reduction in sodium intake reduced blood pressure [51,52]. Finally, pulses (lentils, beans, peas) contain isoflavones [53,54], which possess antiplatelet, anti-atherosclerotic and anti-hypertensive properties [55]. These isoflavonoes, such as anthocyanins, stimulate the secretion of a cardioprotective hormone (i.e., adiponectin) which exerts anti-inflammatory properties in blood vessel cells [56], thus potentially reducing blood pressure and arterial stiffness and improving HRV.

The present study, however, did not find these beneficial effects, which might be due to the difference in participant types and intervention duration. The current study is limited in that it involved healthy individuals, which is not typical of people in hospital settings. People at high risk of CVD might be more sensitive to dietary interventions. Studies involving overweight and obese individuals [57], type 2 diabetic patients [58], older people [23] or people with hypercholesterolemia [59] found significant beneficial effects of pulses on CVD risk factors, whereas in the present study, such beneficial effects were not found in healthy individuals. Six to seven weeks of legume consumption lowers LDL cholesterol in healthy individuals (41 to 78 years) [60]; therefore, the intervention duration might be another factor. Previous interventions lasted from 3 weeks to 12 months (most commonly 3 weeks to 16 weeks), and found reduced risk for CVD via favorable effects on blood lipid profile and blood pressure [49,58,59,61,62,63], whereas the present intervention only lasted 4 days. Hospital bed rest duration ranges from several days to many weeks [64], depending on many factors such as types of diseases, how well the surgery is done, health conditions after surgery, etc. A pulse-based diet may have significant beneficial effects on CVD risk factors in prolonged bed rest. A longer intervention may be needed to investigate the effects of the pulse-based diet.

Our study had several limitations. The major limitation was the small sample size. This study was intended as a proof of principal to support a larger confirmatory study in a hospital setting with a larger number of participants and for a longer time period. Another limitation was participant demographics. All participants included were healthy adults, rather than hospital patients. Future studies are needed to assess the effects of the pulse diet on individuals with specific disease types, e.g., bone fractures, high blood pressure, and type 2 diabetes.

## 5. Conclusions

The importance and practical application of this study is that this will inform the nutrition habits and diets of people who have to undergo bed rest during hospital care, long-term care or at-home recovery. Optimizing the diets and nutritional habits of patients is important for preventing or offsetting negative health implications (e.g., impaired insulin sensitivity, increased insulin resistance and increased bone breakdown), improving other clinical measures, and reducing morbidity levels [6], length of hospital stay [7] and health care costs [7]. The benefits of the pulse diet may be amplified in older participants with long-duration illness who would be exposed to possibly greater CVD risk factors secondary to age and pre-hospital illness that led to the hospital stay.

## Figures and Tables

**Table 1:** Comparison of pulse-based diet and hospital diet.

	Pulse-Based Diet	Hospital Diet
Energy intake (kcal/day)	1016 ± 311	1019 ± 307
Fat (g/day)	28.3 ± 8.7	24.5 ± 11.2
Saturated fat (g/day)	4.9 ± 1.0	5.9 ± 2.3
Carbohydrate (g/day)	152.1 ± 49.1	152.1 ± 41.2
Protein (g/day)	47.8 ± 13.3	45.3 ± 15.0
Fibre (g/day)	25.9 ± 9.4 *	6.7 ± 2.3
Sodium (mg)	730 ± 334 *	1886 ± 718
Energy intake (% energy) per day for 3 meals		
Breakfast (% energy)	20.8 ± 10.7	20.8 ± 10.6
Lunch (% energy)	40.7 ± 19.3	42.6 ± 17.7
Dinner (% energy)	38.5 ± 13.5	36.6 ± 10.9

Values are mean ± SD. * Significantly different from hospital diet, *p* < 0.05.

**Table 2:** Diet and time main effects.

	Hospital Diet			Pulse Diet		
	Pre-Bedrest	Post-Bedrest	Change	Pre-Bedrest	Post-Bedrest	Change
**OGTT**						
Glucose I-AUC (mmol L^−1^ h) *	218 ± 58	308 ± 57	90 ± 52	230 ± 102	358 ± 102	127 ± 196
Glucose T-AUC (mmol L^−1^ h) *	693 ± 77	761 ± 77	68 ± 39	680 ± 109	774 ± 66	95 ± 123
Insulin I-AUC (mU L^−1^ h)	4702 ± 801	5095 ± 2060	393 ± 1408	3725 ± 1625	4086 ± 1203	362 ± 636
Insulin T-AUC (mU L^−1^ h)	5627 ± 905	6070 ± 2253	443 ± 1438	4781 ± 1734	4775 ± 1477	−6.2 ± 458
HOMA-IR *	1.38 ± 0.54	1.37 ± 0.50	−0.01 ± 0.24	1.48 ± 0.54	0.88 ± 0.37	−0.59 ± 0.42 ^a^
Matsuda Index	6.54 ± 1.94	6.39 ± 2.71	−0.14 ± 1.10	7.14 ± 2.36	8.75 ± 3.13	1.61 ± 1.49 ^a^
**Bone resorption**						
Ntx level (Nm BCE) × 10^3^ *	262 ± 175	430 ± 222	168 ± 66	265 ± 75	361 ± 136	96 ± 65 ^a^
Ntx/lean mass (Nm BCE/g) *	6.2 ± 3.6	10.8 ± 4.5	4.6 ± 1.6	6.6 ± 1.7	9.2 ± 2.9	2.6 ± 1.6 ^a^
Ntx/crt (Nm BCE/mg/mL)	4611 ± 4789	3845 ± 1790	−766 ± 3285	2258 ± 1636	3646 ± 2311	1388 ± 909
**Blood lipids**						
Triglyceride (mmol/L)	0.7 ± 0.3	0.7 ± 0.4	0.0 ± 0.3	0.6 ± 0.3	0.6 ± 0.3	0.0 ± 0.3
Total cholesterol (mmol/L)	4.0 ± 0.7	4.2 ± 0.6	0.2 ± 0.4	4.1 ± 0.8	4.2 ± 0.9	0.1 ± 0.6
HDL (mmol/L)	1.6 ± 0.4	1.5 ± 0.4	−0.1 ± 0.3	1.6 ± 0.3	1.6 ± 0.5	0.0 ± 0.3
LDL and VLDL (mmol/L)	2.7 ± 0.7	3.1 ± 1.1	0.4 ± 0.6	2.9 ± 0.8	2.8 ± 1.1	−0.1 ± 0.8
**Body composition**						
BMC (g)	2084 ± 561	2029 ± 498	−55 ± 120	1987 ± 458	2024 ± 528	37 ± 90
Fat mass (kg)	17.0 ± 10.1	17.0 ± 10.0	0 ± 0.4	16.8 ± 9.6	16.8 ± 9.5	0 ± 0.3
Lean mass (kg) *	40.9 ± 11.1	39.2 ± 10.6	−1.7 ± 0.9	41.1 ± 11.8	39.7 ± 11.8	−1.4 ± 0.9
Lean + BMC (kg) *	43.0 ± 11.6	41.3 ± 11.1	−1.8 ± 0.9	43.1 ± 12.3	41.7 ± 12.4	−1.4 ± 0.8
Total mass (kg) *	60.0 ± 15.8	58.3 ± 15.2	−1.7 ± 0.8	59.9 ± 15.5	58.5 ± 15.4	−1.4 ± 0.7
% fat *	27.7 ± 10.9	28.6 ± 11.3	0.9 ± 1.0	27.8 ± 11.2	28.5 ± 11.6	0.7 ± 0.8
BMD (g/cm^2^)	1.12 ± 0.11	1.09 ± 0.09	−0.03 ± 0.04	1.09 ± 0.08	1.10 ± 0.09	0.01 ± 0.02
Trunk fat mass (kg)	7.5 ± 5.2	7.7 ± 4.6	0.1 ± 0.9	7.4 ± 4.7	7.5 ± 4.5	0.1 ± 5.7
Spine BMC (g)	47.9 ± 12.7	48.3 ± 13.3	0.3 ± 1.6	47.8 ± 12.3	48.0 ± 13.3	0.2 ± 1.9
Spine BMD (g/cm^2^)	0.89 ± 0.10	0.89 ± 0.10	0.0 ± 0.03	0.89 ± 0.10	0.90 ± 0.17	0.0 ± 0.01
Total hip BMC (g)	32.6 ± 12.9	33.1 ± 12.5	0.5 ± 1.7	31.6 ± 11.8	32.2 ± 12.3	0.7 ± 0.8
Total hip BMD (g/cm^2^) *	0.94 ± 0.19	0.95 ± 0.18	0.01 ± 0.02	0.93 ± 0.19	0.94 ± 0.19	0.02 ± 0.01
Femoral neck BMC (g)	3.7 ± 1.0	3.9 ± 1.2	0.2 ± 0.2	3.8 ± 1.2	3.9 ± 1.2	0.10 ± 0.2
Femoral neck BMD (g/cm^2^)	0.76 ± 0.16	0.77 ± 0.18	0.01 ± 0.03	0.76 ± 0.19	0.78 ± 0.17	0.02 ± 0.02
**Arterial stiffness and HRV**						
Pulse wave velocity (m/s)	9.4 ± 1.6	9.2 ± 1.6	−0.2 ± 2.2	9.9 ± 2.8	9.2 ± 2.0	−0.7 ± 1.5
SDRR (ms)	55 ± 34	53 ± 31	−2 ± 9	49 ± 32	40 ± 31	−9 ± 9
Heart rate (beats/min)	69 ± 13	66 ± 10	−3 ± 7	67 ± 13	67 ± 13	0 ± 5
SDSD (ms)	47 ± 32	34 ± 14	−13 ± 21	40 ± 30	30 ± 9	−10 ± 25
RMSSD (ms)	47 ± 32	34 ± 14	−13 ± 21	40 ± 30	30 ± 9	−10 ± 25
Prr50 (%)	17 ± 18	14 ± 13	−3 ± 11	18 ± 19	10 ± 9	−7 ± 12
LF Power (%)	31 ± 23	24 ± 7	−7 ± 22	34 ± 12	24 ± 11	−10 ± 12
LF Power (nu)	50 ± 36	56 ± 20	6 ± 24	56 ± 23	45 ± 17	−11 ± 19
HF Power (%)	38 ± 31	22 ± 14	−15 ± 26	29 ± 17	29 ± 12	0 ± 15
HF Power (nu)	48 ± 33	45 ± 19	−3 ± 22	44 ± 22	55 ± 17	11 ± 19
LF/HF *	2.6 ± 2.9	1.8 ± 1.7	−0.8 ± 1.6	2.0 ± 1.9	1.1 ± 1.0	−1.0 ± 1.3

Values are mean ± SD; * Significant time main effects, *p* < 0.05; ^a^ significantly different from hospital diet, *p* < 0.05 Abbreviations: IAUC, incremental area under the curve; TAUC, total area under the curve; HOMA-IR, homeostatic model assessment of insulin resistance; Ntx, N-telopeptides; crt, creatinine, HDL, high density lipoproteins; LDL + vLDL, low density lipoproteins and very low density lipoproteins; BMC, bone mineral content; BMD, bone mineral density; HRV, heart rate variability; SDRR, standard deviation of RR interval; SDSD, standard deviation of successive differences; RMSSD, root mean square of successive differences; Prr50, number of pairs of adjacent RR intervals differing by more than 50 ms to all RR intervals; LF, low frequency; HF, high frequency.

**Table 3:** Blood pressure during the bed rest period.

	Systolic Blood Pressure (mmHg)		Diastolic Blood Pressure (mmHg)	
	Hospital Diet	Pulse Diet	Hospital Diet	Pulse Diet
Day 1	103 ± 10	102 ± 13	65 ± 5	64 ± 7
Day 2	103 ± 9	102 ± 11	64 ± 7	65 ± 6
Day 3	104 ± 10	97 ± 16	66 ± 7	61 ± 9 *
Day 4	103 ± 11	100 ± 15	65 ± 7	63 ± 10

Values are mean ± SD * Significant interaction between diet and time, *p* < 0.05.
